# Azacytidine arrests ripening in cultivated strawberry (*Fragaria* × *ananassa*) by repressing key genes and altering hormone contents

**DOI:** 10.1186/s12870-022-03670-1

**Published:** 2022-06-07

**Authors:** Félix Juan Martínez-Rivas, Rosario Blanco-Portales, Francisco Javier Molina-Hidalgo, José Luis Caballero, Leonardo Perez de Souza, Saleh Alseekh, Alisdair R. Fernie, Juan Muñoz-Blanco, Antonio Rodríguez-Franco

**Affiliations:** 1 Department of Biochemistry and Molecular Biology, University of Cordoba, Edificio Severo Ochoa, Campus de Rabanales, E-14014 Córdoba, Spain; 2 Max-Planck-Institute of Molecular Plant Physiology, Am Mühlenberg 1, 14476 Potsdam-Golm, Germany; 3 Center of Plant Systems Biology and Biotechnology, Ruski Blvd. 139, 4000 Plovdiv, Bulgaria

**Keywords:** Azacytidine, Demethylation, Ripening, m5-cytosine, Abscisic acid

## Abstract

**Background:**

Strawberry ripening involves a number of irreversible biochemical reactions that cause sensory changes through accumulation of sugars, acids and other compounds responsible for fruit color and flavor. The process, which is strongly dependent on methylation marks in other fruits such as tomatoes and oranges, is highly controlled and coordinated in strawberry.

**Results:**

Repeated injections of the hypomethylating compound 5-azacytidine (AZA) into green and unripe *Fragaria* × *ananassa* receptacles fully arrested the ripening of the fruit. The process, however, was reversible since treated fruit parts reached full maturity within a few days after AZA treatment was stopped. Transcriptomic analyses showed that key genes responsible for the biosynthesis of anthocyanins, phenylpropanoids, and hormones such as abscisic acid (ABA) were affected by the AZA treatment. In fact, AZA downregulated genes associated with ABA biosynthetic genes but upregulated genes associated with its degradation. AZA treatment additionally downregulated a number of essential transcription factors associated with the regulation and control of ripening. Metabolic analyses revealed a marked imbalance in hormone levels, with treated parts accumulating auxins, gibberellins and ABA degradation products, as well as metabolites associated with unripe fruits.

**Conclusions:**

AZA completely halted strawberry ripening by altering the hormone balance, and the expression of genes involves in hormone biosynthesis and degradation processes. These results contradict those previously obtained in other climacteric and fleshly fruits, where AZA led to premature ripening. In any case, our results suggests that the strawberry ripening process is governed by methylation marks.

**Supplementary Information:**

The online version contains supplementary material available at 10.1186/s12870-022-03670-1.

## Introduction

Cultivated strawberry (*Fragaria × ananassa*) is one of the most economically important fleshly fruits grown worldwide. The sensory quality of this highly appreciated soft fruit, which is a dietary ingredient of millions of people, is dictated by events that occur largely during ripening [1]. Strawberry development and ripening involves irreversible biochemical and genetic processes leading to the production of an edible, desirable soft receptacle as a strategy for helping the seed dispersal [2]. These changes are governed by various factors such as hormones, temperature, light, and genotype, and are modulated by the expression of a set of ripening related genes. How all these factors interact with one another to mediate the process is currently under extensive research [3, 4].

Hormonal control of strawberry ripening is widely documented. Thus, the auxin/ABA content ratio is known to govern development and ripening of the fruit [2, 5]. However, ethylene, which plays a major role in the ripening of climacteric fruits, has little effect on the ripening of non-climacteric fruits such as strawberry [6]. Auxins, which in strawberries are primarily present as indole 3-acetic acid (IAA), are mainly synthesized in the achenes. Auxin activity, which regulates the expression of many developmental related genes, is mostly associated with receptacle growth and development [3]. Thus, auxin contents peak in fully grown green receptacles (G3 stage) but are decreased in red (R) and senescent (SE) fruits. Therefore, they behave antagonistically with ABA during development and ripening. In fact, immature receptacles with detached or unfertilized achenes fail to grow and ripen. These effects, however, can be reversed by application of exogenous auxin [7]. ABA, which is responsible for ripening, peak once the fruit has fully developed, concomitant with a decline in auxin and gibberellic acid contents [2, 8]. Increased ABA levels elicited by exogenous application or by drought stress lead to premature fruit ripening. Conversely, depleting ABA by silencing or inhibiting the *FaNCED1* gene, which is responsible for its biosynthesis, considerably delays the process [2]. Gibberellic acid is primarily responsible mainly for cell expansion during fruit development; also, it seemingly arrests ripening via controlling of ABA levels in *F. vesca* [9–11].

Ripening in all kinds of fruits is also regulated by epigenetic marks [12, 13]. This is especially true for tomato [14–16], where regulated demethylation of the promoters and bodies of the *Cnr*, *Rin* and *Nor* genes –which are associated with ethylene-dependent ripening– occurs through *SlDMR2* demethylase [15, 16]. However, methylation marks may also be lost through a decreased activity of de novo and maintenance DNA methyltransferases [17]. This seems to be the case with strawberry, where an active decay in RNA directed DNA methylation (RdDM) was found to regulate ripening [18].

DNA methylation patterns can also be altered by hyper- and hypomethylating compounds. For example, 5-azacytidine (AZA) induces general, nonspecific DNA hypomethylation [19]. This compound is a cytidine analog that, after its metabolization to 5-aza-2′-deoxycytidine-triphosphate, replaces cytosine during DNA replication. AZA derived bases cannot be methylated; rather, they bind covalently to DNA methyltransferases, which causes their degradation and eventually reduces methylation of DNA during replication [19, 20].

AZA has been used to elucidate the effect of reduced DNA methylation on fruit development and ripening. Thus, adding 400 μM AZA to cultured orange cells was found to downregulate genes involved in the biosynthesis of carotenoids and upregulate those involved in its degradation. The resulting decrease in carotenoid content —and hence also in ABA contents— led to the loss of the orange color [21]. In another study, spraying 100 μM AZA on grapes caused early ripening by increasing glucose, soluble acids and anthocyanin levels [22]. This was also the case with green tomatoes injected with 1 mM AZA [14, 15]. Similarly, surface application of 1 mM AZA to apples and peaches triggered accelerated ripening and caused the early appearance of red pigments in both fruits [23, 24]. Finally, spraying strawberry fruits with 20 mM AZA also resulted in early ripening [18], whereas injecting fruits with an identical concentration of AZA arrested ripening [25].

This paper reports a striking, unexpected finding: reversibly delayed ripening of strawberry receptacles upon injection of 1 mM AZA. The levels of transcripts and metabolites associated with hormone biosynthesis, aroma, flavor and texture were either negligible or undetectable following AZA treatment. This was also the case with the expression of a number of significant transcription factors (TFs) involved in the ripening process. Key and critical genes pertaining to the ABA biosynthetic pathway were downregulated, whereas other genes involved in ABA degradation were upregulated. AZA treated parts exhibited substantially reduced ABA levels but considerably increased inactive and conjugated ABA levels. Genes involved in auxin and gibberellin biosynthesis were upregulated, and so were the contents of these ripening inhibitory hormones. Injection of the demethylating compound AZA therefore seemingly arrests ripening by inhibiting expression of many key TFs resulting in a misbalance of hormone contents, which suggests a major controlling role of DNA methylation in the process.

## Results

### 5-Azacytidine delays ripening and alters the transcriptome in strawberry

**Fig. 1:**
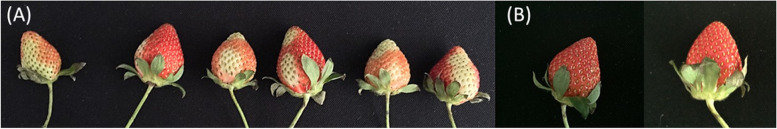
**A** Halves of AZA treated strawberry fruit receptacles. **B** Treated fruits after the AZA treatment was stopped. Fruits were initially collected at the green G3 stage (fully developed green receptacles) and kept with pedicels immersed in a Murashige and Skoog media solution. Then 0.5 mL of a 1 mM solution of AZA was injected into fruit halves every other day. The treated parts remained green or white. The other halves were injected with water and used as controls. They ripened at a regular pace and developed a normal red color

Injecting 1 mM AZA to halves of immature strawberry receptacles completely arrested ripening. Thus, as can be seen in Fig. 1A, no red color formed in the treated halves. The other halves, which were injected with water, ripened at a regular pace and reached the red stage uneventfully. AZA treated parts remained white as far as the treatment was repeated every other day; once it was stopped, however, the treated parts turned red and ripened normally (Fig. 1B). An RNA-Seq analysis performed to identify transcriptome changes in AZA treated tissues relative to control samples revealed that a total of 3275 genes were downregulated and 1685 upregulated (Additional file 1).

### AZA alters expression of transcription factors that regulate ripening

A number of key TFs involved in ripening exhibited decreased transcript levels in AZA treated tissues (Additional file 2). This was especially so for *FaMYB10* (*FvH4_1g22020*), which is a master regulator of the phenylpropanoid pathway through which red pigments are biosynthesized [26] and also for *FaRIF* (*FvH4_3g20700*), knockdown and overexpression of which delays ripening and accelerates the process, respectively [27]. *FaDOF2* (*FvH4_2g14390*) was also expressed at low levels. This TF operates synergistically with *FaEOBII* (*FvH4_6g50930*) to regulate the expression of *FaEGS2* (*FvH4_2g09110*) which encodes eugenol synthase 2 —an enzyme responsible for the biosynthesis of eugenol, which influences the aroma and flavor of this fruit [28, 29]. The TFs *FaGAMYB* (*FvH4_7g04470*) [30] and *FaASR* (*FvH4_2g13410*) [31], silencing of which also delays ripening, were also expressed at low levels. The list of TFs that are usually induced during ripening but downregulated in AZA treated tissues includes *FaMADS9* (*FvH4_6g46420*), which is responsible for the deposition of sugars and waxes during ripening [32], and *FaPRE1* (*FvH4_3g04310)*, silencing of which leads to repression of ripening related genes [33] (Additional file 2). Interestingly, AZA induced expression of *FaMYB9* (*FvH4_2g31130*) and *FaMYB11* (*FvH4_6g34650*) (Additional file 2), two TFs involved in the biosynthesis of proanthocyanins that are mainly accumulated in immature and green receptacles [34].

### AZA affects expression of methylation and demethylation related genes

The expression pattern of a group of genes related with DNA methylation and demethylation, that were previously identified by Gu et al. [35] in response to the AZA treatment, was carefully analyzed. AZA downregulated the DNA methyltransferases, *FaMET1* (DNA methyltransferase, *FvH4_1g10290*) and *FaDRM1.3* (de novo methyltransferase 1.3, *FvH4_6g50980*), expression of which was previously found to increase during ripening in *F. vesca*. Also, AZA markedly decreased expression of *demethylase 3* (*FvH4_2g10170*) and *demethylase 4* (*FvH4_6g07060*) was clearly reduced in response to AZA treatment (Additional file 3).

### AZA alters genes associated with sensory properties in strawberry

Structural genes involved in the anthocyanin pathway such as *FaPAL* (phenylammonia lyase, *FvH4_6g16060*), *Fa4CL* (4-coumaryl-CoA ligase, *FvH4_6g16460*), *FaDFR* (dihydroflavonol reductase, *FvH4_2g39530*), *FaF3H* (flavone 3-hydroxylase, *FvH4_1g11810*) and *FaANS* (anthocyanin synthase, *FvH4_5g01170*) [36] were all downregulated in AZA treated tissues. *FaGT1*, which encodes the essential anthocyanidin glucosyltransferase (*FvH4_7g33480*) [37] and *FaMT1* (*FvH4_6g46740*) [38] –involved in the biosynthesis of pelargonidin-3-glucoside and its malonyl derivative– were expressed at low levels. By contrast, *FaANR* (*FvH4_3g02980*), which encodes an anthocyanin reductase, was expressed at high levels (Additional file 4). This gene is involved in the biosynthesis of proanthocyanins, the main phenylpropanoid compounds present at the green stage [37].

AZA altered the expression of a number of genes associated with changes in sensory attributes such as color, aroma and firmness. Thus, it downregulated some genes coding for enzymes associated with the synthesis of aroma volatiles, including *FaEGS2* (eugenol synthase 2, *FvH4_2g09110*) [28]; the alcohol acyl transferases *FaAAT1* (*FvH4_7g18570*) and *FaAAT2* (*FvH4_3g38880*) [39, 40]; and *FaQR* (quinone reductase, *FvH4_6g50700*) [41] (Additional file 5). AZA had a similar effect on cell wall related genes and decreased the levels of some cell wall modifying enzymes such as *FaRGLyase* (rhamnogalacturonate lyase, *FvH4_1g19200*), *FaPL* (pectate lyase, *FvH4_2g19540*) and *FaPG* (polygalacturonase, *FvH4_7g15040*) [42, 43]. AZA treated fruit parts additionally exhibited increased transcription of genes expressed mainly at the green stages such as xyloglucan endotransglucosylase/hydrolase (*FvH4_5g05180*) and UDP-glucuronate 4-epimerase 1 (*FvH4_5g05180*) (Additional file 5).

### AZA alters the biosynthetic pathways and content balance of hormones

**Fig. 2:**
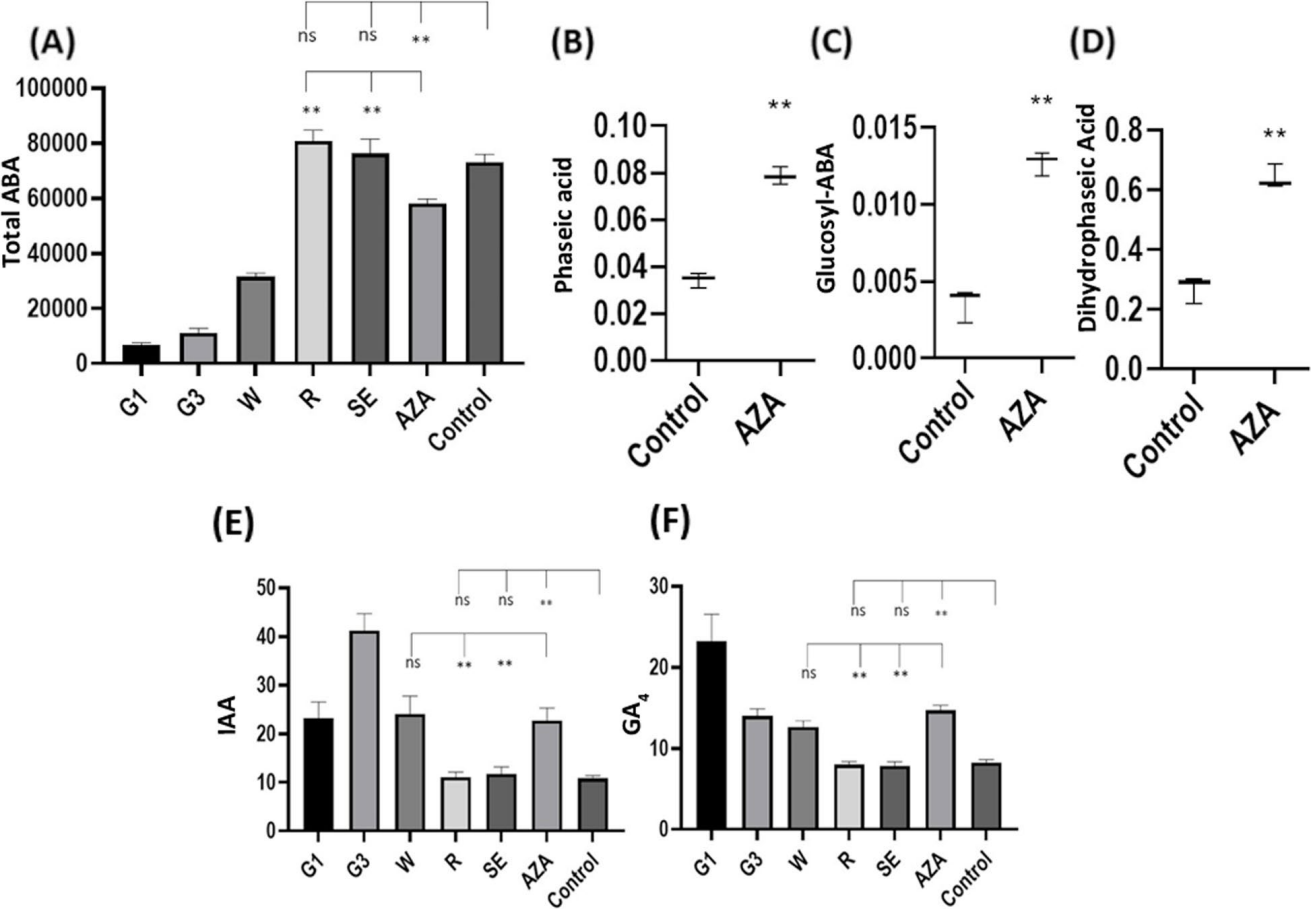
Relative hormone abundance at different stages of strawberry receptacle development and ripening

**Fig. 3:**
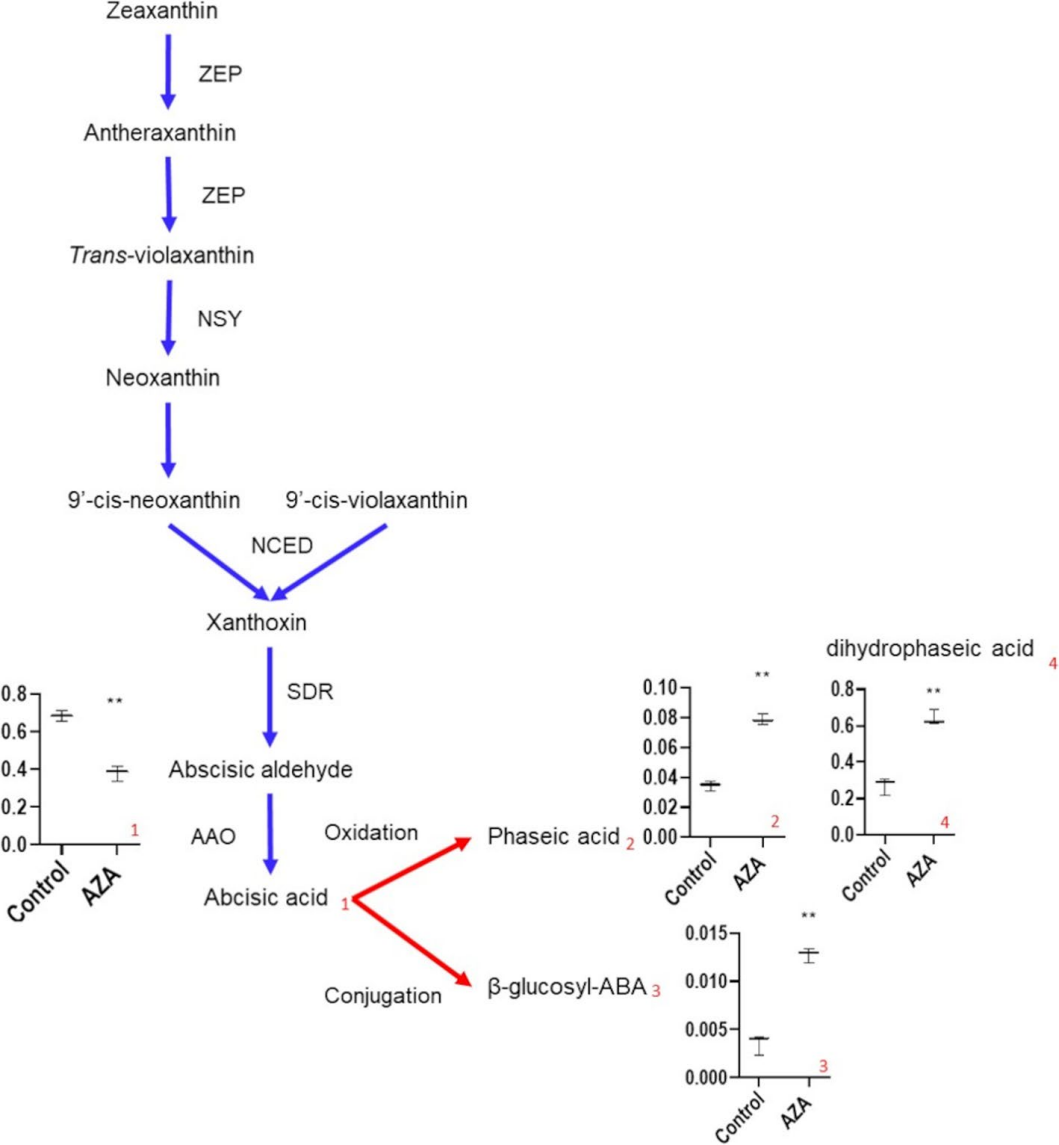
ABA biosynthetic and degradation pathways. The enzymes were down regulated and upregulated by the AZA treatment are shown in blue and red, respectively. Boxplots show the levels of metabolites in control and treated receptacles, the *y*-axis representing the means ± SE of normalized area for each compound as determined for internal standard isovitexin in LC–MS analysis. Three biological replicates were used. ZEP zeaxanthin epoxidase; NSY neoxanthin synthase; NCED 9-*cis*-epoxycarotenoid dioxygenase; SDR Short chain dehydrogenase reductase; AAO Abscisic aldehyde oxidase. Significant differences as determined with Student’s *t*-test analysis (**) *p* < 0.05

AZA markedly downregulated a number of genes involved as ABA precursors in carotenoid biosynthesis including *phytoene synthase* (*FvH4_6g38780*), *zeaxanthin epoxidase* (*FvH4_1g16080*), *neoxanthin synthase* (*FvH4_5g20670*), *short chain dehydrogenase-reductase* (*FvH4_5g01930*) and *abscisic aldehyde oxidase* (*FvH4_3g26980*) (Additional file 6). It also boosted withdrawal of bioactive ABA as AZA increased expression of the enzymes *ABA 8′-hydroxylase* (a cytochrome P450*, FvH4_2g34420*)*,* which causes irreversible ABA degradation, and *ABA β-glucosyltransferase* (*FvH4_2g05690*)*,* which inactivates this hormone through a reversible conjugation reaction. Total (free and conjugated) ABA contents increased during the ripening process and peaked at the red stage, in untreated parts (Fig. 2A). By contrast, AZA treated parts exhibited a 30% reduction relative to untreated, control parts (Fig. 2A). Interestingly, treated parts exhibited increased levels of conjugated ABA, and also of ABA degradation products such as phaseic and dihydrophaseic acid (Fig. 2B-D, Fig. 3).

Auxins are synthesized via two different pathways involving genes that were highly expressed in AZA treated tissues. The transcript levels of key enzymes for auxin biosynthesis such as *Trp decarboxylase I* (*FvH4_3g09540*), *amidase I* (*FvH4_2g01730*) and *YUCCA10* (*FvH4_2g24750*) were also highly expressed in AZA treated parts, and so were the auxin transporters *FaPIN1* (*FvH4_5g17310*), *FaPIN4* (*FvH4_4g32450*), and *FaPIN5* (*FvH4_6g00660*), and the genes involved in auxin conjugation (Additional file 6). Injecting AZA into strawberry fruits did not cause auxin levels to drop as in the control tissues; rather such levels remained similar to those observed at the white stages (Fig. 2E).

AZA also upregulated some genes associated with gibberellin biosynthesis such as *gibberellin-3-beta-dioxygenase I* (*FvH4_2g30040*) and *IV* (*FvH4_3g36530*), and also some gibberellin receptors (*FvH4_2g06540, FvH4_2g06530*) (Additional file 6). Gibberellin contents peaked at the G1 stage, decreased at the G3 and W stages, and were even lower at the red and senescent stages (Fig. 2F). Gibberellin levels in AZA treated parts exceeded those in untreated fruits at white stages, whereas those in the control samples were similar to the contents in untreated samples at red stage (Fig. 2F).

### AZA induces metabolic differences

A comprehensive metabolic profile revealed differences between AZA treated and control samples. Thus, AZA considerably raised the levels of a number of amino acids that usually decrease during ripening (Phe, Ile, Lys, Asn, Pro and Thr, mainly; Table 1). However, it decreased sucrose levels roughly by one half as the potential result of its reducing the transcription levels of *sucrose-6-phosphate synthase* (*FaSPS, FvH4_2g04920*, *FvH4_2g28820*), *sucrose synthase* (*FaSUS*, *FvH4_2g26000*) and the *sucrose transporter* (*FaSUT*, *FvH4_5g33660*) (Additional file 7). There were, however, no significant changes in fructose or glucose levels (Table 1).

**Table 1:**
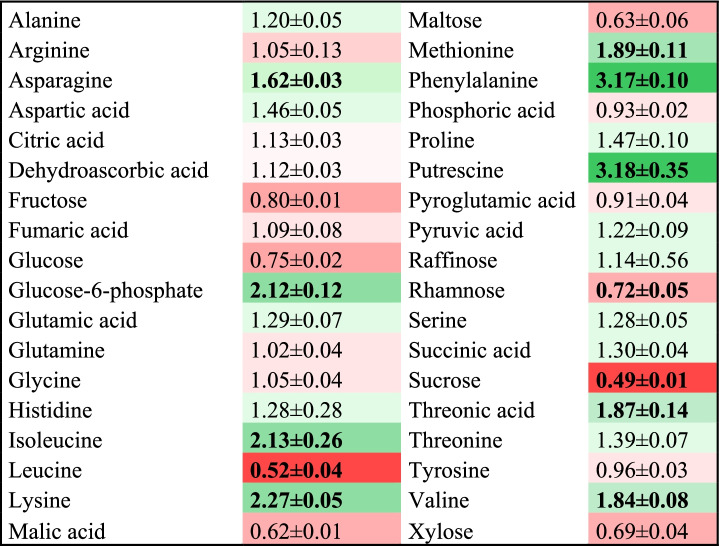
Changes in primary metabolites in AZA treated parts relative to the control

Data are normalized to the mean response calculated for internal standard ribitol. Values presented are fold change means ± SE of three biological replicate each versus control. Values in bold denote significant differences as determined with Student’s *t-*test analysis (*p* < 0.05). Colors indicate the proportional content of each identified metabolite in the samples. Low and high values are depicted in cells of differently shaded red and green color, respectively

Major soluble phenolics in AZA treated and control tissues were identified by Ultra Performance Liquid Chromatography-Mass spectrometry (UPLC–MS). As expected, anthocyanins such as pelargonidin-3-glucose, which is the main source of the red color of strawberry, and pelargonidin-3-(6′-malonylglucoside), pelargonidin-3-rutenoside, pelargonidin-3,5-diglucoside, cyanidin-3-glucose, cyanidin-3-(6′malonylglucoside) and cyanidin-3-rutenoside, were either detected at negligible levels or were not detected in AZA treated samples (Table 2). Major flavonols typically present in strawberry receptacle (e.g., kaempferol, quercetin derivatives) were found at decreased levels in AZA treated samples (Table 2), which additionally exhibited increased levels of pelargonidin and cyanidin dimers and trimers, and also of proanthocyanins (Table 2). These compounds are synthesized and accumulated at the green stages and then catabolized to produce the anthocyanin monomers that accumulate during ripening [34]. The building blocks of proanthocyanins, (epi)afzelechin and epi(catechin), were also present at increased in treated parts (Table 2). Taken together, the previous results suggest that the metabolic and transcriptomic profiles of AZA treated parts were similar to those found at the white and immature stages of ripening.

**Table 2:**
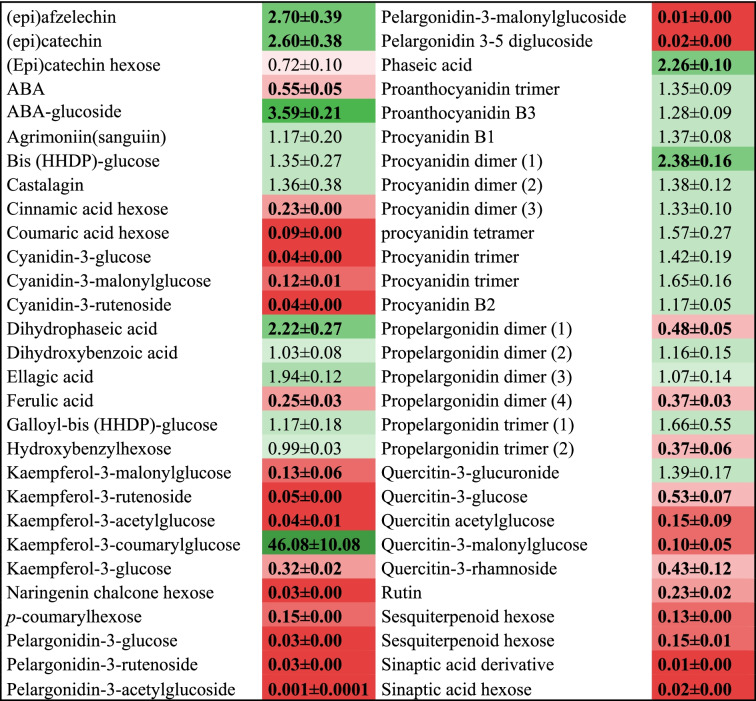
Changes in secondary metabolism in AZA treated parts relative to the control

Data are normalized to the mean response calculated for internal standard isovitexin. Values presented are fold change means ± SE of three biological replicates each versus control. Values in bold denote significant differences as determined with Student’s *t*-test analysis (*P* < 0.05). Colors indicate the proportional content of each identified metabolite in the samples. Low and high values are depicted in cells of differently shaded red and green color, respectively

## Discussion

### AZA-mediated overall demethylation arrests ripening in strawberry fruit

Fleshly fruits tissues seemingly respond differently to AZA depending on its concentration, and on how and when it is applied. Thus, applying AZA to tomato surface activates phytoene synthase expression, and hence ripening [14]. Surface application of AZA to apples and peaches has similar effects; namely, it activates the phenylpropanoid pathway and raises anthocyanin contents as a result [23, 24]. This is also the case with strawberries, where surface spraying of immature fruits with 20 mM AZA resulted in their early ripening [18] but direct injection of 1 mM AZA into receptacles had the opposite effect. Delayed ripening was also observed following injection of 20 mM AZA into unripen strawberry fruits [25]. Other fruits similarly treated with AZA exhibit identical response patterns. This concentration (1 mM) was used as a result of a screening with different concentrations resulting in the same halted phenotype and according to other reported fruits treatments, as tomato [15] and peach [23]. Here, AZA injection completely halted ripening; in fact, the white stage lasted for as long as receptacles were injected every other day (Fig. 1). A lack of colour development was previously observed also in AZA treated orange callus tissue [21].

AZA is known to have a wide range of effects on a variety of biological systems depending on its dose, application method and exposure time [44]. Therefore, encountering contrasting responses is unsurprising given the complex regulation of developmental processes that are affected by DNA methylation. Demethylation by AZA is presumably a chaotic process whereby a number of genes related with ripening may be induced or repressed. Thus, surface spraying strawberries with 20 mM AZA induced premature ripening, while injecting the same concentration or lower concentration (1 mM) used in this work, delayed ripening. Injection reaches all tissues and is thus a more direct application method than surface spraying is. Indeed, the latter may lead to water soaking fruit damage and not penetrating the fruit due to wax layer [45]. Many other aspects should be considered when attempting to explain these differences, including the use of a different cultivar, the in vivo vs in vitro treatment, greenhouse vs growth chamber, constant or variable temperature. But at this point, we can only speculate and more work would be needed to elucidate it. These findings open up new avenues for further research.

Treated tissues remained white by effect of AZA downregulating a number of key ripening related TFs such as *FaMYB10, FaGAMYB, FaSHP* and *FaRIF.* Silencing of these genes by RNAi delayed ripening and caused fruits to remain white, similarly to the AZA treatment [26, 27, 30, 46] (Additional file 3). That the outcome is a complete cessation of the ripening process rather than a mere reduction of red color development is supported by the fact that many other ripening related genes such as those influencing sensory attributes of the fruit were downregulated. Such genes included some involved in aroma (*FaAAT1* and *FaAAT2*) [39, 40], volatile acid biosynthesis (*FaDOF2, FaEOBII* and *FaEGS2*) [28, 29] and cell wall softening (*FaPG* and *FaRGLyase*) [43, 47] (Additional files 4, 5).

Strawberry development and ripening are governed by the ABA/auxin concentration ratio through exquisitely controlled biosynthesis of these hormones in space and time. Indeed, a misbalance or alteration in this ratio can stop or accelerate these processes [31]. Under normal conditions, ABA levels rise during ripening and peak in fully red ripe receptacles [8] (Fig. 2A). By contrast, other hormones present at early development stages such as gibberellin and auxins reach higher concentrations at the green and white stages [8]. However, application of AZA caused a misbalance in hormone levels. Thus, AZA treated parts had high auxin and gibberellin contents typical of the green and white stages, and ABA contents about 30% lower than those in red, ripe receptacles. Hormone levels in the control samples, which were treated with water, were similar to those found in red untreated fruits (Fig. 2A, E, F). High auxin levels are known to arrest strawberry ripening [7] and to block ABA biosynthesis [2].

An interlinked regulatory loop between ABA, gibberellin and auxin levels in strawberry receptacles has been proposed [11] suggesting that auxins help to maintain high gibberellin levels in receptacles at early development stages. Gibberellins repress *FaNCED,* which is required for ABA biosynthesis, but induces FaCYP707A4a*,* an enzyme involved in ABA degradation. In this situation, ripening is arrested. Once receptacles grow in full, auxins and gibberellin levels drop, and bioactive ABA accumulates, and as a result ripening is triggered. A similar response involving downregulation of NCED enzymes and decreased ABA levels was previously found in AZA treated citrus callus [21]. Here, AZA treated tissues had ABA, auxin and gibberellin levels similar to those in untreated green and white fruits, which explains why they remained unripe.

**Fig. 4:**
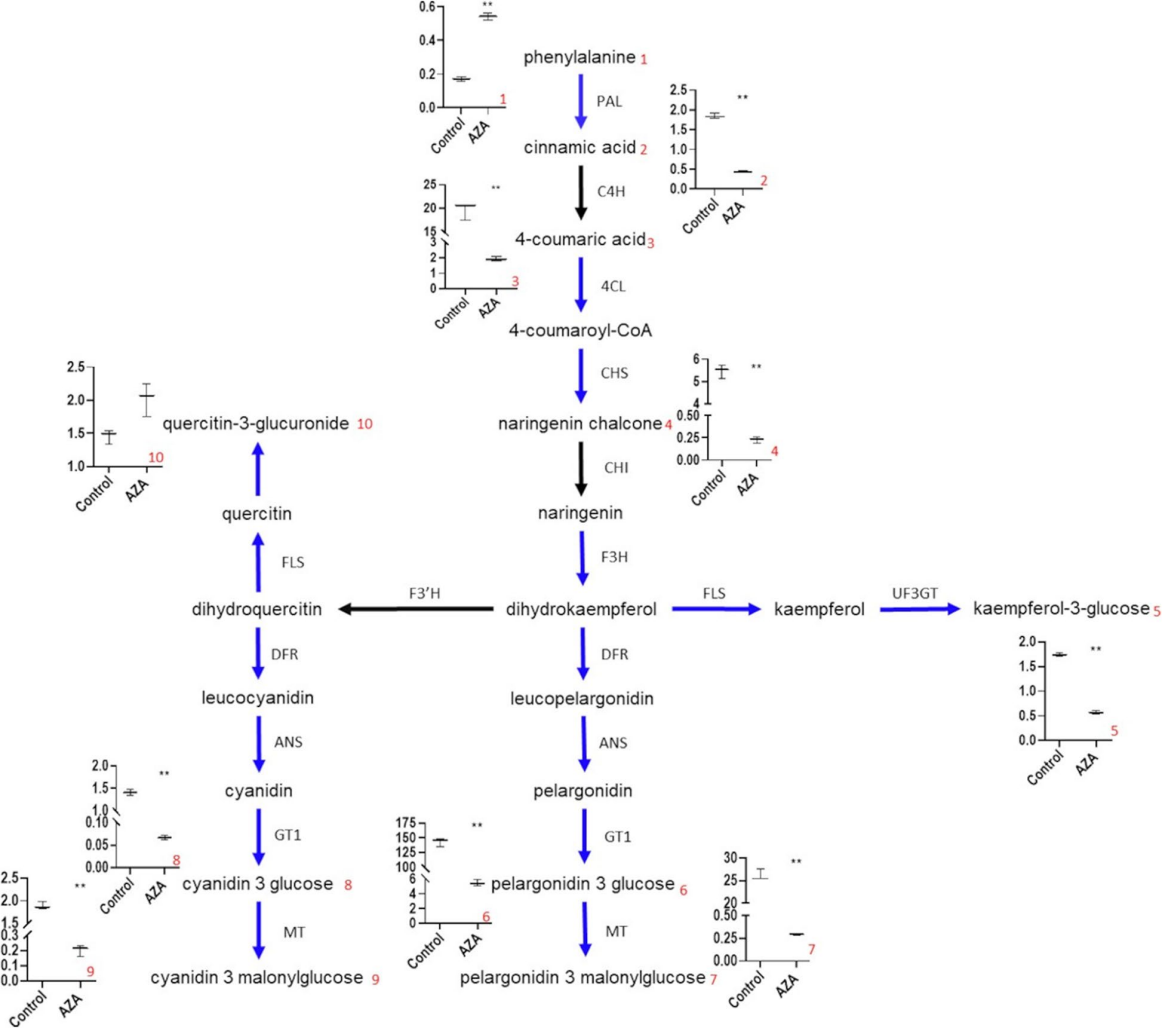
Scheme of the phenylpropanoid, flavonoid and anthocyanin pathways. The enzymes down-regulated by the AZA treatment are shown in blue. Boxplots show the levels of metabolites in control and treated receptacles, with the *y*-axis representing the normalized area of each compound normalized to internal standard isovitexin and fresh weight in LC–MS analysis. Three biological replicates were used. PAL phenylammonia lyase; 4CL 4-coumaryl-CoA ligase; ANS anthocyanidin synthase; C4H cinnamic acid 4-hydroxylase; CHI chalcone isomerase; CHS chalcone synthase; DFR dihydroflavonol reductase; F3H flavanone 3-hydroxylase; F3’H flavonoid3’-hydroxylase; GT1 anthocyanin 3-glucosyltransferase; MT malonyl transferase; UF3GT UDP flavonol 3–glucosyltransferase. Significant differences as determined with Student’s *t*-test (**) *p* < 0.05

The levels of amino acids, putrescine and glucose-6-phosphate decrease during ripening [6, 32]. These compounds were found at higher levels in AZA treated parts than in control samples (Table 1). Red pigmentation is conferred mainly by pelargonidin-3-glucoside and its derivatives, which accumulate in receptacles throughout ripening. The phenylpropanoid pathway has been studied in depth and most of the enzymes involved have been identified [36, 37]. During the process, phenylpropanoid and flavonols are present at low concentrations in unripe fruits but accumulate in red and mature receptacles. Proanthocyanidins and condensed tannins are mainly present at green stages [34]. AZA treated tissues contained increased amounts of proanthocyanins and condensed tannins, but decreased amounts of anthocyanin and flavonols (Table 2), the decrease was correlated with reduced expression of genes that encodes enzymes involved in the biosynthesis of phenylpropanoids and flavonols such as *FaANS* (anthocyanidin synthase), *Fa3GT* (anthocyanin glucosyltransferase) and *FaMT1* (anthocyanin malonyl transferase) (Fig. 4, Additional file 4). Anthocyanins were also present at low levels in *FaGAMYB*, *FaMYB10* and *FaRIF* RNAi silenced fruits [26, 27, 30]. These TFs were all markedly downregulated by AZA. As a result, the metabolomic profile of AZA treated tissues resembled that observed at early ripening stages.

Sucrose was recently deemed a major regulator of strawberry fruit ripening [31, 48]. Adding sucrose to strawberry fruits induced ripening by activating *FaNCED1* [48]*.* This was accompanied by repression of genes responsible for ABA withdrawal such as ABA-glucosyltransferases. RNAi silencing of the *FaSUT1*, gene, which encodes a sucrose transporter, causes strawberry fruits not to ripen [48]. Interestingly, AZA treated tissues exhibited downregulated expression of *FaSUT1* and substantially reduced sucrose levels here (Additional file 7). Thus, genes directly activated by sucrose signaling such as *FaASR* and *FaNCED1* were clearly downregulated in treated tissues and prevented sucrose signaling from promoting ripening.

In tomato, a decay in total DNA 5mC levels occurs at the onset of ripening concomitantly with timely, specific directed demethylation of a group of key, specific regulatory genes required for the fruit to ripen. The effect is mediated by ripening-specific demethylases such as SlDML2 [16]. Overall demethylation also occurs at the onset of ripening in strawberry; however, it does not seem to be mediated by specific demethylases even though there is a decay in the de novo and maintenance RdDM methylation pathway [18]. Although there were no expression changes in the RdDM pathway in response to AZA, there was marked downregulation of DNA methyltransferases (*FaMET1* and *FaDRM1.3*; Additional file 3), which suggests that AZA may cause a loss of DNA methylation marks. Also, there was decreased expression of *demethylase 3* and *4*, the functional regulatory roles of which remain unknown.

Although methylation is widely believed to inhibit gene expression, some recent studies suggest that methylation induce it [49, 50]. Thus, some TFs require methylation of their promoters in order to physically bind to DNA and attract the transcriptional machinery. DNA methylation is an active field of work and its role in the gene expression regulation is rather complex. Further research is therefore needed to clarify these results and gain deeper insight into the underlying molecular mechanisms.

## Conclusions

AZA altered the expression of genes responsible for the production of high levels of auxin and gibberellins. Also, it decreased ABA levels by repressing those genes affecting its biosynthesis and inducing those responsible for its withdrawal. The resulting hormone misbalance led to repression of a number of TFs involved in the ripening process. In fact, many genes coding for the biosynthesis of pigments, aroma and cell wall modifying enzymes that depend on these regulatory TFs were expressed at low or negligible levels. Although the response pattern of strawberry contradicts those for other fruits such as tomato, control of the ripening process may involve methylation marks also in the former. Thus, methylation marks in strawberry probably govern the balance in the auxin/ABA ratio, which has been deemed a key point for ripening control.

## Materials and methods

### Plant material

Strawberry plants (*Fragaria × ananassa* Duch. cv. Fortuna, an octoploid and commercial cultivar) were grown under open field conditions at the “El Cebollar” experimental station (Moguer, Huelva, SW Spain). Full sized fruits at the green stage were harvested alongside their pedicels, and immersed in Murashige and Skoog medium. Fruits were kept in a plant chamber at 25 °C with a 16/8 h light/dark period. Fruits were harvested at various developmental stages and classified as follows: small-sized green fruits (G1, 2–3 g), full-sized green fruits (G3, 4–7 g), white fruits (W, 5–8 g), full-ripe red fruits (R, 9–15 g) overripe fruits (OR, 9–15 g) and senescent fruits (SN, 9–15 g), and immediately frozen in liquid nitrogen for transport and storage at − 80 °C until use. All samples were obtained by agreement with the management of the experimental station.

### 5-Azacytidine treatments

5-Azacytidine (AZA, Sigma–Aldrich Cat no. A2385) was dissolved at a 1 mM concentration in sterile water. The resulting solution was passed through 0.22 μm polyethersulfone filters and aliquots of approximately 0.5 mL were carefully injected into green strawberry receptacles by using an HPLC syringe whose needle was inserted around 0.3–0.5 cm into the fruits without reaching the pith. This concentration is identical with those previously used in other fruits such as tomato [15] and peach [23]. One half of each fruit was injected with AZA and the other with sterile water for use as a control in order to minimize differences among gene variants in the fruits. AZA injections were repeated every other day for three to five times until the water injected half of each fruit was fully ripe. Then, the two halves were split with a scalpel, frozen in liquid nitrogen and stored at − 80 °C until use. Achenes, the true seeds, were carefully removed from the surface of frozen fruit receptacles with a scalpel.

### RNA extraction, RNA-seq and analysis of differentially expressed genes (DEGs)

Total RNA was isolated and purified from three independent biological replicates, using a previously reported method [51]. Total RNA isolated was treated with DNase (RNase free; Invitrogen) according to the manufacturer’s instructions. RNA samples were deemed DNA-free when no amplicons corresponding to the 26S–18S interspacer were detected from PCR reactions. Gene expression analyses for RNA-Seq validation were performed by quantitative real-time PCR (qRT-PCR) with an iCycler device (Bio-Rad) as described elsewhere [26]. The relative increase or decrease in gene expression was calculated as described elsewhere [52], using interspacer 26S–18S as control gene on the grounds of its constitutive expression. The primers used are listed in Additional file 8.

Libraries were compiled by using Illumina’s TruSeq Stranded mRNA Library Prep Kit according to the manufacturer’s instructions. Briefly, each sample was enriched in mRNA by selecting 3’poly(A) tails from poly(T) magnetic beads. Captured mRNAs were converted into cDNA and sequencing adaptors were added to their ends to make the molecules ready for sequencing. Samples were dual indexed for post-sequencing demultiplexing. The fragment size distribution and concentration of the libraries were checked by using the Agilent DNA 1000 kit on an Agilent 2100 Bioanalyzer. They were sequenced as 2x100b paired-end sequences on a HiSeq-4000 Illumina platform. A total of 50 ± 10 million reads were obtained library which corresponded to approximately 40–50X coverage of the total haploid genome size. These were analyzed with FastQC and trimmed with Trimmomatic when required to remove low-quality bases and/or adapters. Reads were then mapped to the fasta coding sequences corresponding to version 4.0.a1 of the *Fragaria vesca* genome [53] by using Kallisto v. 0.46.1. Differential expression was then analyzed by using the DESeq2 package in R after importing the data with tximport. A threshold cutoff (− 2 ≤ log_2_FoldChange ≥ 2) was set for differentially expressed genes and adjusted to *p* ≤ 0.01. The fasta sequences corresponding to the coding sequences for each gene were downloaded from the Rosaceae web page (http://bit.ly/2uSHd2n). Sequences and mapped data were deposited in the public GEO database GSE151534. Additional file 1 shows the whole RNA-Seq results, and Additional file 9 the validation of the RNA-Seq results via qRT-PCR.

### Metabolite and hormone determinations

#### Primary metabolites

Primary metabolites were extracted and determined by gas chromatography mass spectrometry as described elsewhere [54]. Briefly, frozen ground material from three biological replicates was homogenized in 300 μL of methanol at 70 °C for 15 min, followed by 200 μL of chloroform and 300 μL of water. The polar fraction was dried under vacuum and the residue was derivatized in 40 μL of 20 mg mL^− 1^ methoxyamine hydrochloride in pyridine at 37 °C for 30 min, followed by 70 μL of MSTFA at 37 °C for 30 min. A Multi-Purpose autosampler from Gerstel GmbH & Co.KG, was used to inject samples into a gas chromatograph interfaced to a time-of-flight mass spectrometer (viz., a Pegasus HT TOF-MS instrument from LECO Corporation). Helium at a constant flow rate of 2 mL/s^− 1^ was used as a carrier gas and gas chromatography performed on a 30 m DB-35 column. The injection temperature was 230 °C, and those of the transfer line and ion source were both 250 °C. The initial temperature of the oven (85 °C) was raised 15 °C/min^− 1^ to a final temperature of 360 °C. After a solvent delay of 180 s mass spectra over the *m*/*z* range 70–600 were recorded at a rate of 20 scans s^− 1^. Mass chromatograms were evaluated by using the software packages Chroma TOF 4.5 from Leco and TagFinder 4.2.

#### Secondary metabolites

A previously reported protocol [55] was used to profile secondary metabolites on a Waters Acquity UPLC system coupled to a Q-Exactive Orbitrap mass detector. The UPLC system was equipped with an HSS T3 C18 reversed phase column (100 × 2.1 mm i.d., 1.8-μm particle size; Waters) operated at 40 °C. The mobile phase consisted of 0.1% formic acid in water (Solvent A) or acetonitrile (Solvent B) as was circulated at a flow rate of 400 μL min^− 1^. The injected volume was 2 μL. The UPLC instrument interfaced connected to an Exactive Orbitrap from Thermo Fisher Scientific via a heated electrospray source from the same manufacturer. Spectra were recorded in the full scan positive and negative ion detection mode other the *m/z* range 100–1500. The resolution was set at 25000 and the maximum scan time at 250 ms. The sheath gas was set to a value of 60, while the auxiliary gas was set to 35. The transfer capillary and heater temperature used was 150 and 300 °C, respectively. The spray, capillary and skimmer voltage was 3, 25 and 15 V, respectively. Mass spectra were recorded from minute 0 to 19 of the UPLC gradient. Molecular masses, retention times and associated peak intensities were extracted from the raw files by using RefinerMS v. 5.3 from GeneData, and Xcalibur from Thermo Fisher Scientific. Metabolites were identified and annotated by using standard compounds, literature and strawberry metabolomics references [32, 56]. All data are reported in standard-compliant formats [57].

#### Hormones

About 200 mg of fresh-frozen material from three biological replicates was used to extract and purify hormones as previously described elsewhere [58] except for the following minor modifications: the material was extracted with 80% acetonitrile containing 1% acetic acid (10 mL) at 4 °C for 1 h. After centrifugation at 3000 *g* for 20 min and collection of the supernatant, the residue was re-extracted with the same solvent and centrifugated. Then, acetonitrile was evaporated and the residue dissolved in 2 mL of water containing 1% acetic acid, followed by sonication for a few minutes. The aqueous residue was loaded onto an OASIS HLB cartridge (60 mg, 3 cm^3^; Waters) and prewashed/equilibrated consecutively with 3 mL of acetonitrile, methanol and 1% aqueous acetic acid. After washing with 1% aqueous acetic acid (3 mL), hormones were eluted with 80% acetonitrile containing 1% acetic acid (2 × 3 mL). Once dried, the eluate was dissolved in 700 μL of water containing 1% acetic acid and passed through a SepPak silica cartridge (100 mg; Waters) that was prewashed with MeOH (2 × 700 μL) and 0.1 M HCl (700 μL). The extract was loaded onto an equilibrated MCX column, eluted with acetonitrile and evaporated and the residue was dissolved in 700 μL of water containing 1% acetic acid. The aqueous residue was loaded onto a Wash WAX solid phase column after prewashing with MeOH and then 0.1 M NaOH, followed by equilibration with water. The column was then washed with MeOH and eluted by 80% acetonitrile containing 1% acetic acid, the eluate being evaporated and the residue dissolved in 30 μL of water containing 1% acetic acid. Hormones were quantified with the UPLC Triple Quad MS system, following a protocol described elsewhere [58]. Statistical significance was assessed with a Student’s *t*-test as implemented in Graphpad v. 8.0.

## Supplementary Information

**Additional file 1.**

**Additional file 2.**

**Additional file 3.**

**Additional file 4.**

**Additional file 5.**

**Additional file 6.**

**Additional file 7.**

**Additional file 8.**

**Additional file 9.**

## Data Availability

The datasets reported herein be found in GEO repository. https://www.ncbi.nlm.nih.gov/geo/query/acc.cgi?acc=GSE151534

## Abbreviations

ABA: Abscisic acid
AZA: 5-azacytidine
DNA: Deoxyribonucleic acid
TF: Transcription factor
UHPLC—MS: Ultra High Performance Liquid Chromatography–Mass Spectrometry
