# Supplementary material for: Azacytidine arrests ripening in cultivated strawberry (Fragaria × ananassa) by repressing key genes and altering hormone contents
Source: BMC Plant Biol. 2022 Jun 7;22:278. doi: 10.1186/s12870-022-03670-1 (PMC9172142; doi:10.1186/s12870-022-03670-1)
Supplement: Supplementary file 9 — Additional file 9. [file 12870_2022_3670_MOESM9_ESM.docx]

**Additional file 9:** Expression data of selected genes in AZA treated tissues obtained by qRT-PCR. All genes were statistically significant with respect to the reference sample (Control) as determined by the student’s t-test p <0.05
